# Herbivores alter plant–wind interactions by acting as a point mass on leaves and by removing leaf tissue

**DOI:** 10.1002/ece3.3249

**Published:** 2017-07-27

**Authors:** Adit R. Kothari, Nicholas P. Burnett

**Affiliations:** ^1^ Department of Integrative Biology University of California Berkeley CA USA

**Keywords:** biomechanics, fluttering, herbivory, plant–wind interactions, reconfiguration

## Abstract

In nature, plants regularly interact with herbivores and with wind. Herbivores can wound and alter the structure of plants, whereas wind can exert aerodynamic forces that cause the plants to flutter or sway. While herbivory has many negative consequences for plants, fluttering in wind can be beneficial for plants by facilitating gas exchange and loss of excess heat. Little is known about how herbivores affect plant motion in wind. We tested how the mass of an herbivore resting on a broad leaf of the tulip tree *Liriodendron tulipifera*, and the damage caused by herbivores, affected the motion of the leaf in wind. For this, we placed mimics of herbivores on the leaves, varying each herbivore's mass or position, and used high‐speed video to measure how the herbivore mimics affected leaf movement and reconfiguration at two wind speeds inside a laboratory wind tunnel. In a similar setup, we tested how naturally occurring herbivore damage on the leaves affected leaf movement and reconfiguration. We found that the mass of an herbivore resting on a leaf can change that leaf's orientation relative to the wind and interfere with the ability of the leaf to reconfigure into a smaller, more streamlined shape. A large herbivore load slowed the leaf's fluttering frequency, while naturally occurring damage from herbivores increased the leaf's fluttering frequency. We conclude that herbivores can alter the physical interactions between wind and plants by two methods: (1) acting as a point mass on the plant while it is feeding and (2) removing tissue from the plant. Altering a plant's interaction with wind can have physical and physiological consequences for the plant. Thus, future studies of plants in nature should consider the effect of herbivory on plant–wind interactions, and vice versa.

## 1 INTRODUCTION

Wind is an important abiotic component of many terrestrial habitats, and it can exist over a wide range of spatial and temporal scales (de Langre, 2008). For trees, long‐term wind patterns can affect the overall growth by processes such as prompting growth of reaction wood or altering the structure of root systems and branches (Coutts & Grace, 1995; Goodrich, Ortiz, & Coughlin, 2016; Stokes & Guitard, 1997). On the other hand, short‐term periods of faster‐than‐normal wind can cause tree mortality by up‐rooting or breaking the tree (Mitchell, 2012). Even for leaves on trees, anomalously fast or slow wind can have physical and physiological consequences (Vogel, 2009). Fast wind can apply aerodynamic forces (i.e., drag) that prematurely dislodge the leaves from a tree or damage the leaves by tearing and ripping (Vogel, 1989), and slow wind can result in leaves heating up to temperatures that are physiologically stressful (Roden & Pearcy, 1993b; Vogel, 2009).

Leaf motion is closely associated with many of the stresses induced by wind. Flexible leaves can reduce the drag they experience in wind by reconfiguring into a stable, streamlined shape (e.g., a cone) and reducing their projected areas (Albayrak, Nikora, Miler, & O'Hare, 2012; Miller et al., 2012; Vogel, 1989). The drag on a leaf can also increase with the amplitude of fluttering, which is closely tied to the size of vortices shed by the leaf (Miller et al., 2012). Lastly, the stability and fluttering of a leaf can be affected by the orientation of the leaf relative to wind (Tadrist, Julio, Saudreau, & de Langre, 2015) and the morphology of the leaf, such as with broad leaves that flutter more readily in slow wind before reconfiguring into streamlined cones in fast wind (Albayrak et al., 2012; Hoerner, 1965; Vogel, 1989). The fundamental physical interactions between the leaves and wind are important to understand because they can affect how the entire plant responds to wind and how the plant might be affected by other stressors in its environment (Vogel, 2009).

Invertebrate herbivores that live on plants span a wide range of body masses, from less than 1 g to more than 10 g, and can represent a significant mass increase for the individual structures on the plant (e.g., leaves, branches), especially when multiple herbivores co‐occur on the same plant (Lindroth, Arteel, & Kinney, 1995; Nijhout & Williams, 1974; Pearson, Pearson, & Ralph, 2006). Additionally, herbivores can modify the structure of a leaf by consuming the leaf's tissues. Despite the common occurrence of herbivores on plants, as well as the physical and physiological effects of leaf motion on the plant, little is known on how herbivores affect passive leaf motion in wind (e.g., de Langre, 2008).

Fundamental principles of mechanics can allow for basic predictions of how herbivory will affect leaf motion in wind. In the simplest case, an herbivore on a fluttering leaf can be modeled as a cantilever beam, the tip of which moves up and down with a frequency that is inversely proportional to the combined mass of the leaf and the herbivore (French, 1971). Therefore, leaves with large herbivores can be expected to flutter at slower frequencies than leaves with small herbivores, and especially leaves that have had tissue consumed by herbivores. Herbivore position on the leaf is also expected to affect flutter: The leaf will flutter at a frequency that is inversely proportional to the distance of the herbivore from the leaf's petiole (i.e., where the leaf connects to a branch) (Macho‐Stadler, Elejalde‐García, & Llanos‐Vázquez, 2015). That is, a leaf with an herbivore close to its distal end (e.g., the tip of the leaf) is expected to flutter more slowly than a leaf with an herbivore near the petiole. Even in the absence of wind, the mass of an herbivore, and its position on the leaf, can alter the spatial orientation of the leaf. If the herbivore produces a twisting moment (proportional to the herbivore's mass and distance from the petiole) that exceeds the stiffness of the leaf and its petiole, the leaf will bend toward the ground, altering its orientation to the wind (Gere & Timoshenko, 1990). These mechanical relationships suggest that the presence of an herbivore, and the damage it inflicts on the plant, will alter the leaf's motion in wind, yet little empirical evidence exists for the effects of herbivores on plant–wind interactions.

**Figure 1:**
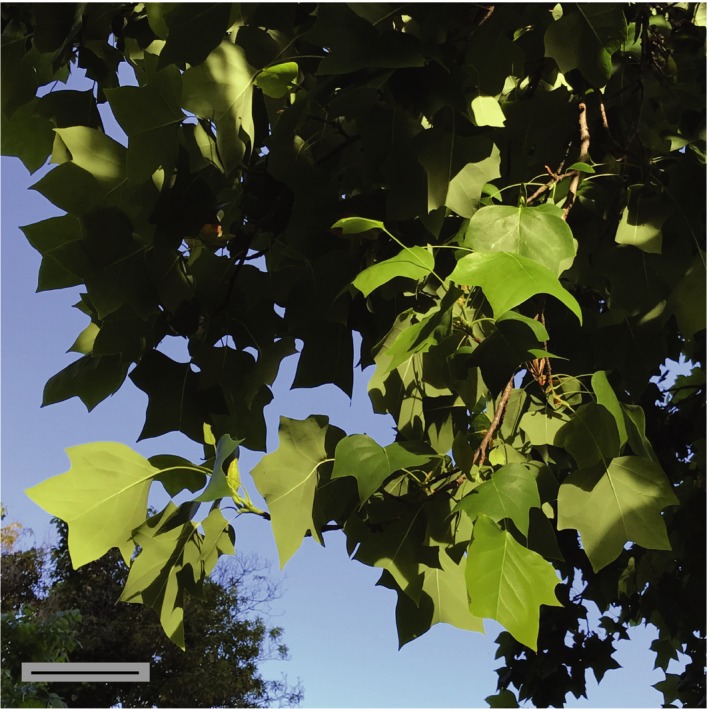
Leaves of the tulip tree *Liriodendron tulipifera*. The scale bar is approximately 10 cm

In the present study, we used leaves of the tulip tree *Liriodendron tulipifera* (Linnaeus) (Fig. 1), a broad‐leafed plant whose interactions with wind are well studied, to examine the effect of herbivores on plant–wind interactions. The leaves of *L*. *tulipifera* characteristically fold into a small, streamlined cone when in fast wind speeds, reducing the drag on the whole leaf. In slow wind, the leaves remain unfolded and more readily flutter vertically, up and down, or horizontally, side‐to‐side (Vogel, 1989). We used *L. tulipifera* to answer the following questions: (1) How does the additional mass of an herbivore affect leaf motion in wind? (2) How does the location of an herbivore on a leaf affect that leaf's motion in wind? (3) How does herbivore damage to a leaf affect that leaf's motion in wind?

## 2 MATERIALS AND METHODS

### 2.1 Sample collection

Leaves of the tulip tree *L. tulipifera* were haphazardly collected from the lowest branches of five individuals on the UC Berkeley Campus, Berkeley, California, USA (37°52′19.76″ N, 122°15′35.13″ W) between August and November 2015. For the experiments that manipulated herbivore mass and position on the leaf, we collected leaves that were free of preexisting herbivore damage. During each collection, approximately three leaves with intact petioles were taken from each tree and then randomly assigned to a treatment group for each experiment. The lengths and planform areas of the collected leaves were measured to the nearest 0.1 cm and 0.01 cm^2^, respectively, with a digital photograph in ImageJ software (v. 1.49b, National Institutes of Health, USA). Leaves that showed signs of wilting were not included in the experiments, following Vogel (1989), and all leaves were used within 3 hours of collection.

**Figure 2:**
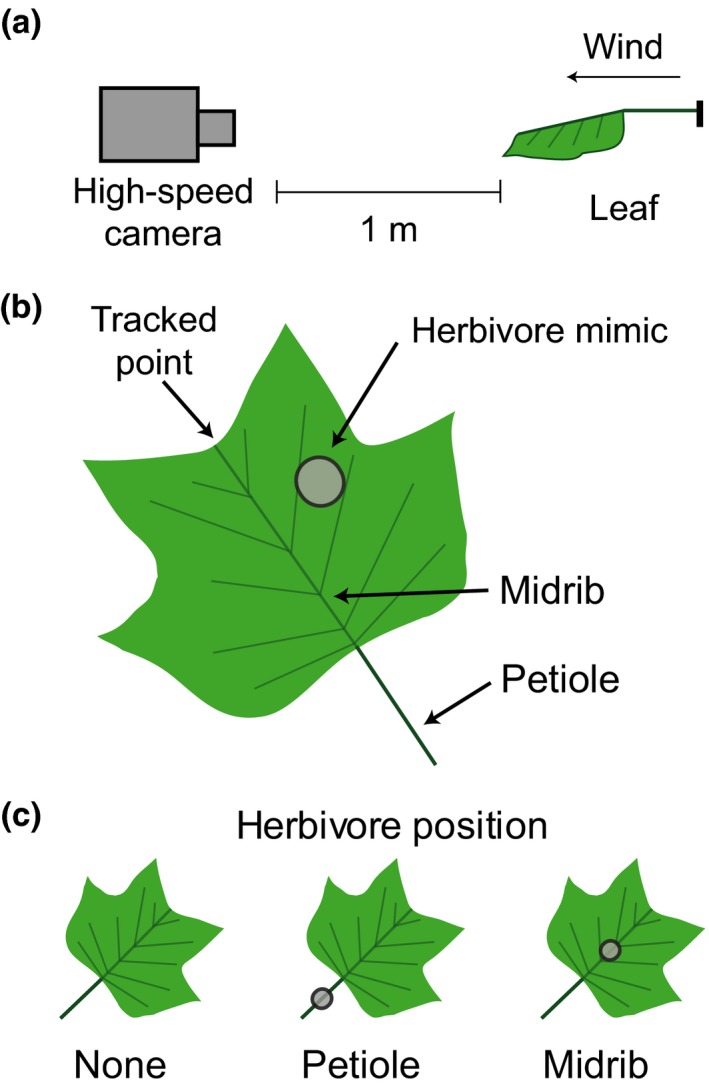
(a) Orientation of the high‐speed camera to achieve an end‐on view of the leaf in the wind tunnel, (b) position of the herbivore mass on the *Liriodendron tulipifera* leaf used when testing how herbivore mass affected leaf movement, and (c) positions of herbivore mimics used when testing how herbivore position affected leaf movement

All experiments were conducted in an open‐jet wind tunnel (opening dimensions: 38 × 38 cm) with nonlaminar wind at two speeds, 1 and 5 m/s, and the movements of leaves were videotaped from an end‐on view at 600 frames per second (Fastec Imaging, San Diego, CA, USA). The camera was positioned at least 1 m from the leaf to minimize parallax (Fig. 2a). Recording began 10 s after the wind tunnel was turned on, and recording lasted between 1 and 10 s. Each leaf experienced both wind speeds, but the order of the wind speeds was randomly assigned for each leaf. The petiole of each leaf was secured in a 2‐cm‐tall clamp on a horizontal beam (diameter = 0.9 cm) in the middle of the wind tunnel's opening, with the petiole parallel to the direction of wind and the horizon, such that wind travelled from the petiole to the distal end of each leaf. The proximal‐most 2 cm of the petiole was wrapped in tape to prevent tissue damage by the clamp. To maintain geometric similarity among all the tested leaves, the length of the unobstructed petiole between the leaf and the clamp was always 40% of the leaf length. The resulting relative length of petiole was within the range of natural petiole lengths (relative to the total leaf area) found on the trees at the collection site. The distal tip of each leaf was marked with a small dot of white paint.

#### 2.1.1 Herbivore mass on leaf

To test whether additional point masses on *L. tulipifera* leaves affected the movements of the leaves in wind, metal weights were glued to a consistent location on each leaf (Fig. 2b). Point masses ranged from 0.0 (no mass) to 11.0 g, covering the range of an additional point mass that can occur with a single invertebrate herbivore and numerous large herbivores. The large mass density of the point masses (4,550 kg/m^3^) allowed us to use smaller sized weights, minimizing any effect that the shape of the weight had on the leaves' motions.

#### 2.1.2 Mass position on leaf

To test whether the location of an herbivore on *L. tulipifera* leaves affected leaf movement in wind, we glued an herbivore mimic (mass = 1.30 g, cylindrical dimensions = 30.0 mm length × 7.5 mm diameter) to one of two positions on the *L. tulipifera* leaves: (1) in the middle of the petiole and (2) on the middle of the leaf along the midrib. Leaves without herbivore mimics were included as controls (Fig. 2c).

#### 2.1.3 Herbivore damage on leaf

To test how herbivore damage to *L. tulipifera* leaves affects the leaves' movement in wind, we collected additional leaves that had preexisting leaf damage. Leaves were selected if they had any amount of missing leaf area but that had an intact midrib.

### 2.2 Leaf movement parameters

Movement of the marked point on the distal tip of each leaf was tracked in ImageJ (see Fig.S1) and used to calculate the following parameters: leaf pitch, mean and maximum fluttering amplitudes, angular direction of leaf movement, dominant and mean fluttering frequencies, and leaf reconfiguration. To remove the effect of leaf translation from leaf fluttering (i.e., capturing just the oscillatory movement of the leaf), we used a linear regression to de‐trend the time series of each leaf's motion. Although the leaf tip can be approximated as moving on the shell of a sphere (with a radius equal to the length of the midrib and petiole), the movement of the leaf was only tracked in the horizontal (*x*) and vertical (*y*) directions. We ignored movement of the leaf in the longitudinal (*z*) direction because movement in the *x* and *y* directions, and the resulting amplitudes, was much greater in magnitude than movement in the *z* direction. The net position of the leaf was calculated as the overall position of the leaf tip in each frame of the video, accounting for both the horizontal and vertical components:

(1) $$\text{Net postion},\;N_{xy}=\sqrt{x^{2}+y^{2}}$$

where *x* and *y* are the horizontal and vertical positions of the leaf tip, respectively. The origin for the horizontal and vertical positions of the leaf tip in each video was an arbitrary pixel in the video frame. Position data were ultimately used to calculate metrics of leaf motion that were not affected by the origin position.

#### 2.2.1 Leaf pitch

In each frame, the angular deflection of the leaf from the horizontal axis was calculated by:

(2) $$\text{Pitch}{(}^{\circ })={\text{sin}}^{-1}\frac{H}{L}$$

where *H* is the vertical distance between the distal tip of the leaf and the clamp holding the petiole and *L* is the length of the leaf midrib and petiole. The pitch calculations from each frame were averaged across the entire video. A negative pitch indicates the leaf is below the horizon.

#### 2.2.2 Fluttering amplitude

The mean amplitudes of fluttering were calculated as:

(3) $$\text{Mean fluttering amplitude},\;A_{\mathrm{mean}}=2\sigma \left(P\right)$$

where σ(*P*) is the standard deviation of time series of position *P* (the *x*,* y*, or *N*_*xy*_ position in each frame) for each leaf. The maximum amplitudes of fluttering were calculated as the total range of each leaf's motion in the *x*,* y*, and *N*_*xy*_ directions, measured during the first half of each video and again during the second half of each video. The two measurements were averaged to give a representative measurement of the maximum amplitude of leaf movement.

#### 2.2.3 Direction of leaf movement

The angular direction of leaf movement was calculated as

(4) $$\text{Flutter direction},\;D_{\mathrm{flutter}}\;{(}^{\circ })={\text{tan}}^{-1}\frac{S_{y}}{S_{x}}$$

where *S*_*x*_ and *S*_*y*_ are the mean fluttering speeds of the leaf in the *x* and *y* directions, respectively, calculated as:

(5) $$\text{Fluttering speed},\;S_{P}=\sqrt{{\left(\frac{\Delta P}{\Delta t}\right)}^{2}}$$

where *P* is the *x* or *y* position in each frame, and *t* is time (s). The fluttering speed was averaged across the entire video. As an example of flutter direction, a value of 0° indicates that the leaf is moving entirely in the horizontal direction, whereas a value of 90° indicates the leaf is moving entirely in the vertical direction.

#### 2.2.4 Fluttering frequency

For leaves that showed a distinct temporal pattern of movement in the horizontal or vertical directions, the dominant frequency of fluttering was calculated as:

(6) $$\text{Dominant fluttering frequency},\;f_{\mathrm{dominant}}\;\left(\text{Hz}\right)=\frac{T}{N}$$

where *N* is the number of complete flutters in a given time period and *T* is the duration of that time period. The mean frequency of fluttering was calculated as

(7) $$\text{Mean fluttering frequency},\;f_{\mathrm{mean}}\;\left(\text{Hz}\right)=\frac{S_{P}}{A_{\mathrm{mean}}}$$

where *A*_mean_ is given by equation (4) and *S*_*P*_ is the mean fluttering speed given by equation (5). Fluttering frequencies were only measured for movement in the *x* and *y* directions.

#### 2.2.5 Leaf reconfiguration

The reshaping or folding of the leaf was calculated as

(8) $$\text{Reconfiguration},\;R_{\mathrm{leaf}}=\frac{{\text{Area}}_{\mathrm{projected}}}{{\text{Area}}_{\mathrm{total}}}$$

where Area_projected_ is the area of the leaf exposed to the wind (i.e., visible to the video camera) and Area_total_ is the total area, or planform area, of the leaf when laid flat. Leaf reconfiguration was measured in four frames of each video, evenly distributed across the entire video, and the values for each frame were averaged to give a mean value of reconfiguration. The measurement of reconfiguration given here is different from that described by others (i.e., the Vogel number) (Albayrak et al., 2012; Miller et al., 2012; Vogel, 1989). The Vogel number quantifies the cumulative effect that streamlining and changing shape have on the drag experienced by a leaf in wind, and it is measured across a range of wind speeds. The definition of reconfiguration presented here (eq. (8)) is solely a measure of the shape change that the leaf undergoes, without a measure of the resulting drag.

All values of leaf movement were calculated relative to the length of the leaf's midrib (i.e., fluttering speed = midrib lengths/s, flutter amplitude = midrib lengths). All calculations and statistical tests were performed with R Statistical Software (v. 3.2.2, Vienna, Austria).

## 3 RESULTS

We characterized the masses and sizes of *L. tulipifera* leaves from our study site. The mass of *L. tulipifera* leaves varied linearly with leaf area and ranged between 0.01 and 0.02 g/cm^2^ (wet weight per leaf area), with a mean mass of 0.02 g/cm^2^ (*SD *< 0.01, *n* = 40). The only parameters of leaf motion that were correlated to leaf size were the fluttering frequencies. In both wind speeds, the dominant horizontal fluttering frequencies of leaves without herbivores or herbivore damage were negatively correlated to the leaf length (linear regression, *p *<* *.05, *n* = 14). However, the mean fluttering frequencies in the horizontal and vertical directions were only correlated with leaf length in 5 m/s wind (linear regression, *p *<* *.05, *n* = 14).

### 3.1 Herbivore mass on leaf

Leaves included in this experiment ranged in midrib length from 4.0 to 12.9 cm (mean = 9.3 cm, *SD *= 1.5, *n* = 56) and leaf planform area from 17.51 to 171.38 cm^2^ (mean = 119.15 cm^2^, *SD *= 29.44). We accounted for variation in leaf size by calculating the relative herbivore mass on each leaf (i.e., herbivore mass per leaf area). This relative herbivore mass (hereafter herbivore load) ranged from 0.00 to 0.05 g/cm^2^. For statistical analyses, we grouped leaves by their herbivore load in bins of 0.01 g/cm^2^.

**Figure 3:**
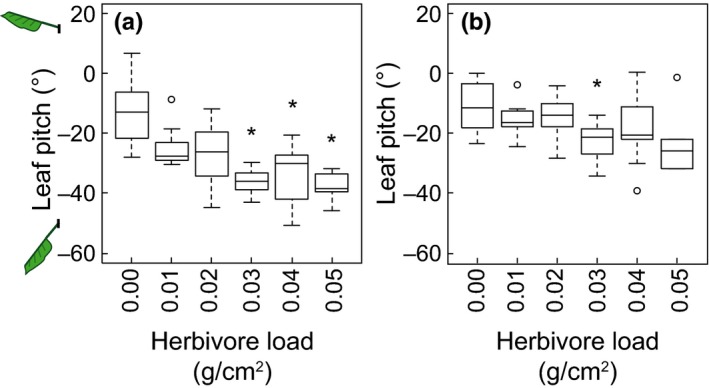
The leaf pitch for each group of herbivore load (herbivore mass per leaf area) in 1 m/s wind (a) and 5 m/s wind (b). Sample sizes for the groups are 14, 7, 10, 11, 9, and 5 for herbivore loads 0.00, 0.01, 0.02, 0.03, 0.04, and 0.05 g/cm^2^, respectively. Asterisks indicate groups whose pitches were significantly different from the pitch of leaves without an herbivore load (Kruskal–Wallis tests with post hoc Dunn tests, *p *< .05, *df *= 5)

In both wind speeds (1 and 5 m/s), the orientation, or pitch, of the leaves decreased as the herbivore load increased (linear regressions of the means of each group of herbivore load; wind = 1 m/s: *y *=* *−457.79*x*−17.21, *p *<* *.05, *r* ^2^ = .83; wind = 5 m/s: *y *=* *−215.20*x*−11.94, *p *<* *.05, *r* ^2^ = .66, *x* is the herbivore load and *y* is the pitch in degrees). In wind of 1 m/s, leaves with herbivore loads of 0.03, 0.04, and 0.05 g/cm^2^ had a significantly lower pitch than leaves without an herbivore load (Kruskal–Wallis test with post hoc Dunn tests, *p *<* *.05, *df* = 5). The median pitches of leaves with herbivore loads of 0.03, 0.04, and 0.05 g/cm^2^ were −35.96°, −30.03°, and −38.45°, respectively, whereas the median pitch of leaves without an herbivore load was −12.80° (Fig. 3a). In wind of 5 m/s, only leaves with herbivore loads of 0.03 g/cm^2^ (median = −21.29°) had a significantly lower pitch than leaves without an herbivore load (median = −11.36°) (Fig. 3b). All leaves, regardless of herbivore load, increased their pitch, or became more horizontal, as wind speed increased from 1 to 5 m/s (paired *t* test, *p *<* *.005, *df* = 55).

At both wind speeds, there were no differences among the herbivore loads for the mean or maximum amplitudes of fluttering in any direction (Kruskal–Wallis tests, *p *>* *.05, *df* = 5). However, all leaves fluttered with a larger amplitude in the horizontal, vertical, and net directions when in 5 m/s than they did in 1 m/s wind (Table 1) (paired *t* tests, *p *<* *.05, *df* = 55). There were also no differences in the angular direction of fluttering among the herbivore loads in either wind speed (Kruskal–Wallis tests, *p *>* *.05, *df* = 5), but leaves in the faster wind fluttered in a more‐horizontal direction than leaves in the slow wind (Table 1) (paired *t* tests, *p *<* *.05, *df* = 55).

**Table 1:** Median and interquartile range (I.Q.R.) for motion between wind speeds for metrics that were not affected by herbivore load (*n *= 56 for each metric)

Motion	Wind speedDirection	1 m/s		5 m/s	
		Median	I.Q.R.	Median	I.Q.R.
Mean amplitude (midrib lengths)	Horizontal	0.06	0.04–0.45	0.15	0.10–0.32
	Vertical	0.04	0.02–0.23	0.14	0.09–0.36
	Net	0.05	0.03–0.22	0.14	0.08–0.25
Maximum amplitude (midrib lengths)	Horizontal	0.15	0.09–0.76	0.33	0.21–0.63
	Vertical	0.11	0.07–0.41	0.35	0.21–0.62
	Net	0.13	0.08–0.38	0.34	0.22–0.57
Direction of fluttering (°)	N/A	52.56	49.30–58.45	46.54	40.24–49.87

**Figure 4:**
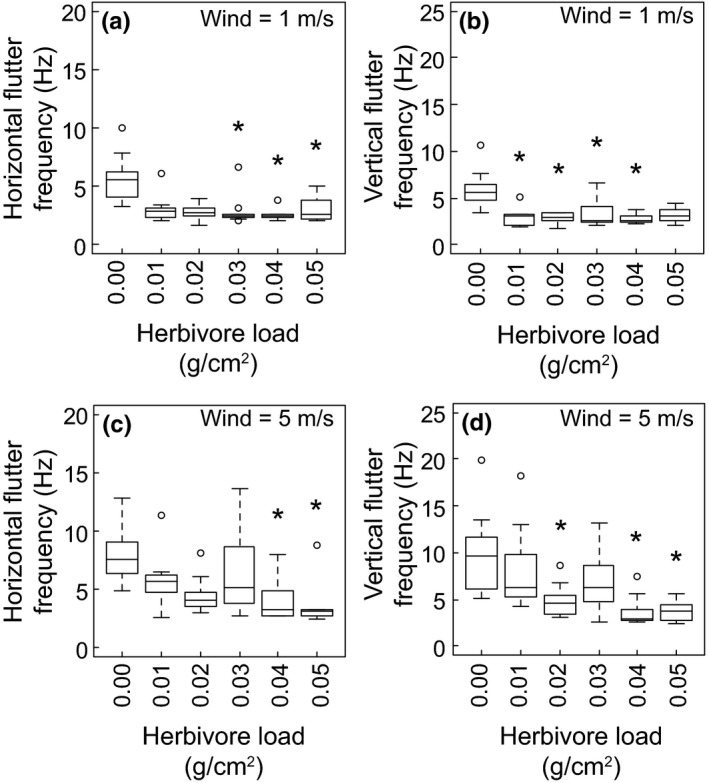
The dominant flutter frequencies of leaves in the horizontal (a) and vertical (b) directions in 1 m/s wind, and the dominant flutter frequencies of leaves in the horizontal (c) and vertical (d) directions in 5 m/s wind. Asterisks indicate that groups are significantly different from leaves without an herbivore load in the same wind speed (Kruskal–Wallis tests with post hoc Dunn tests, *p *< .05, *df *= 5)

Leaves with large herbivore loads fluttered at a slower dominant frequency in the horizontal and vertical directions. In 1 m/s wind, leaves with herbivore loads of 0.02, 0.03, and 0.04 g/cm^2^ fluttered at a slower frequency (medians = 2.68, 2.47, and 2.43 Hz, respectively) in the horizontal direction than leaves without an herbivore load (median = 5.57 Hz) (Fig. 4a). Leaves with herbivores loads of 0.01, 0.02, 0.03, and 0.04 g/cm^2^ fluttered at slower dominant frequencies (medians = 3.02, 2.91, 2.50, and 2.62 Hz, respectively) in the vertical direction than leaves without an herbivore load (median = 5.63 Hz) (Fig. 4b). Similarly, in 5 m/s wind, leaves with herbivore loads of 0.04 and 0.05 g/cm^2^ fluttered at slower dominant frequencies (medians = 3.23 and 3.19 Hz, respectively) in the horizontal direction than leaves without an herbivore load (median = 7.59 Hz) (Fig. 4c). Leaves with herbivore loads of 0.02, 0.04, and 0.05 g/cm^2^ fluttered at slower dominant frequencies (medians = 4.59, 2.94, and 3.76 Hz, respectively) in the vertical direction than leaves without an herbivore load (median = 9.71 Hz) (Fig. 4d). All leaves increased their dominant frequency of fluttering in the horizontal direction and in the vertical direction when wind speed increased from 1 to 5 m/s (paired *t* test, *p *<* *.05, *df *= 51).

The mean frequency of fluttering, a product of the mean fluttering speed and mean fluttering amplitude (eq. (7)), showed less variation with herbivore load than the dominant frequency of fluttering. In 5 m/s wind, leaves with herbivore loads of 0.05 g/cm^2^ fluttered at a slower mean frequency (median = 19.20 Hz) in the horizontal direction than leaves without an herbivore load (median = 29.65 Hz) (Kruskal–Wallis test with post hoc Dunn test, *p *<* *.05, *df *= 5), but there were no differences in mean fluttering frequencies, for either horizontal or vertical fluttering, among the other herbivore loads at each wind speed. For leaves with herbivore loads of 0.05 g/cm^2^, the mean frequency of fluttering in the horizontal direction was faster in the 5 m/s^1^ wind than in the 1 m/s^1^ wind. However, for all other leaves and directions of fluttering, there were no differences in the mean fluttering frequencies between the two wind speeds (paired *t* tests, *p *>* *.05, *df *= 55).

**Figure 5:**
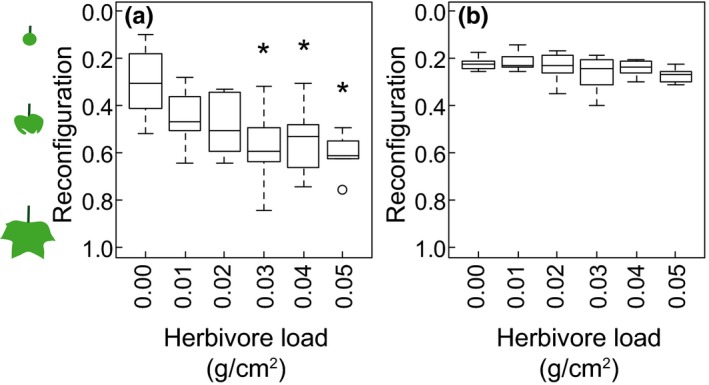
The reconfiguration (i.e., a leaf's projected area divided by its total area) in 1 m/s wind (a) and 5 m/s wind (b). Asterisks indicate groups whose reconfigurations were significantly different from those of leaves without an herbivore load (Kruskal–Wallis tests with post hoc Dunn tests, *p *< .05, *df *= 5)

In both wind speeds, heavier herbivore loads interfered with leaves reconfiguring into smaller projected areas. That is, the median reconfiguration decreased linearly with increasing herbivore load (linear regression of median for each group of herbivore load; wind = 1 m/s: *y *=* *5.18*x *+ 0.37, *p *<* *.05, *r* ^2^ = .71; wind = 5 m/s: *y *=* *0.79*x *+ 0.22, *p *<* *.05, *r* ^2^ = .70). More specifically, in 1 m/s wind, leaves with herbivore loads of 0.03, 0.04, and 0.05 g/cm^2^ were less reconfigured (medians = 0.59, 0.53, and 0.61, respectively) than leaves without an herbivore load (median = 0.30) (Fig. 5a). In 5 m/s wind, there were no significant differences in reconfiguration among the various herbivore loads despite the overall linear trend, described above, for heavier herbivore loads to interfere with reconfiguration (Fig. 5b). All leaves, regardless of herbivore load, were able to reconfigure into smaller projected areas when the wind speed increased from 1 to 5 m/s (paired *t* test, *p *<* *.05, *df* = 55).

### 3.2 Herbivore position on leaf

Leaves used in this experiment ranged in midrib length from 5.9 to 10.8 cm (mean = 7.8 cm, *SD* = 1.3, *n* = 21) and planform area from 42.55 to 120.76 cm^2^ (mean = 81.91 cm^2^, *SD* = 20.29). There were no differences in leaf planform area or leaf length among the three herbivore positions (see Fig. S2). The mean herbivore mass per leaf area was 0.02 g/cm^2^ (*SD* < 0.01) and ranged from 0.01 to 0.03 g/cm^2^. At both wind speeds, there were no effects of herbivore position on leaf pitch, fluttering amplitudes in any direction, angular directions of fluttering, mean fluttering frequencies in either direction, or reconfiguration (Kruskal–Wallis tests, *p *>* *.05, *df* = 2). As wind speed increased from 1 to 5 m/s, leaf pitch, mean amplitude of vertical fluttering, the maximum amplitude of vertical and net fluttering, and the mean frequency of horizontal fluttering all increased (paired *t* tests, *p *<* *.05, *df* = 20). As with the previous experiment, the median direction of leaf movement in 1 m/s wind was 55.80° from horizontal (I.Q.R. = 46.93–63.43), and it decreased significantly in 5 m/s wind to 45.42° from horizontal (I.Q.R. = 42.45–52.68, *n* = 21) (paired *t* test, *p *<* *.05, *df* = 21). Regardless of herbivore position, all leaves were able to reconfigure into smaller projected areas when they were in 5 m/s wind than when they were in 1 m/s wind (paired *t* test, *p *<* *.05, *df* = 21).

**Figure 6:**
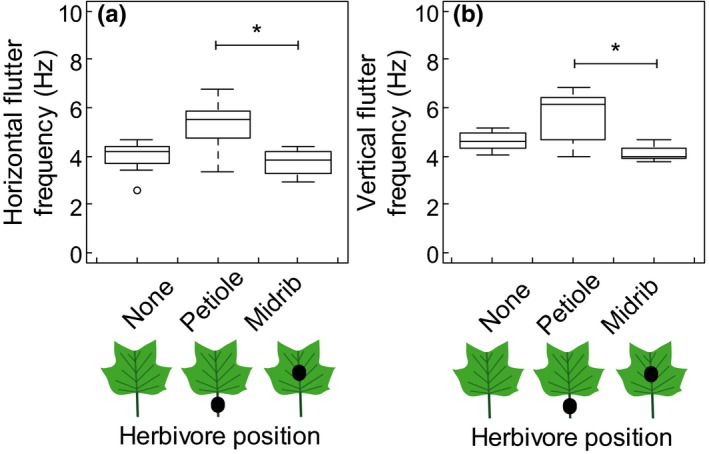
Dominant fluttering frequencies of leaves in the horizontal (a) and vertical (b) directions in 1 m/s wind. Asterisks and brackets indicate groups with significantly different fluttering frequencies (Kruskal–Wallis tests with post hoc Dunn tests, *p *< .05, *df *= 2). Sample size for each group = 7

Herbivore position affected the dominant frequencies of fluttering. In 1 m/s wind, leaves with an herbivore on the petiole fluttered at a faster dominant frequency in the horizontal direction (median = 5.48 Hz) and vertical direction (median = 6.17 Hz) than leaves with an herbivore on the midrib (median = 3.86 and 4.00 Hz for horizontal and vertical fluttering, respectively) (Fig. 6). As with many of the other leaf movement parameters, the dominant frequencies of fluttering in the horizontal and in the vertical directions increased when wind speed increased from 1 to 5 m/s (paired *t* tests, *p *<* *.05, *df* = 21).

### 3.3 Herbivore damage on leaf

The midrib lengths of undamaged leaves (ranging from 4.3 to 9.5 cm, mean = 7.6, *SD* = 1.6, *n* = 21) and the midrib lengths of damaged leaves (ranging from 3.3 to 10.2 cm, mean = 6.9, *SD *= 2.4, *n* = 13) were similar (*t* test, *p *>* *.05). The relative amount of leaf planform area removed by herbivores increased with the leaf length (see Fig. S3). For example, compared to a leaf of the same length, a leaf with length = 10 cm was missing approximately 45% of its area while a leaf with length = 5 cm was missing only 30% of its area. Further details of the herbivore damage are given in Table S1.

There were no effects of herbivore damage on the leaf pitch, fluttering amplitudes in any direction, or the angular direction of leaf movement in either wind speed (Mann–Whitney *U*‐tests, *p *>* *.05). Regardless of herbivore damage, the leaf pitch and all fluttering amplitudes increased in magnitude as wind speed increased from 1 to 5 m/s (paired *t* tests, *p *<* *.05, *df *= 33). However, the angular direction of fluttering did not change with wind speed (paired *t* test, *p *>* *.05, *df* = 33).

**Figure 7:**
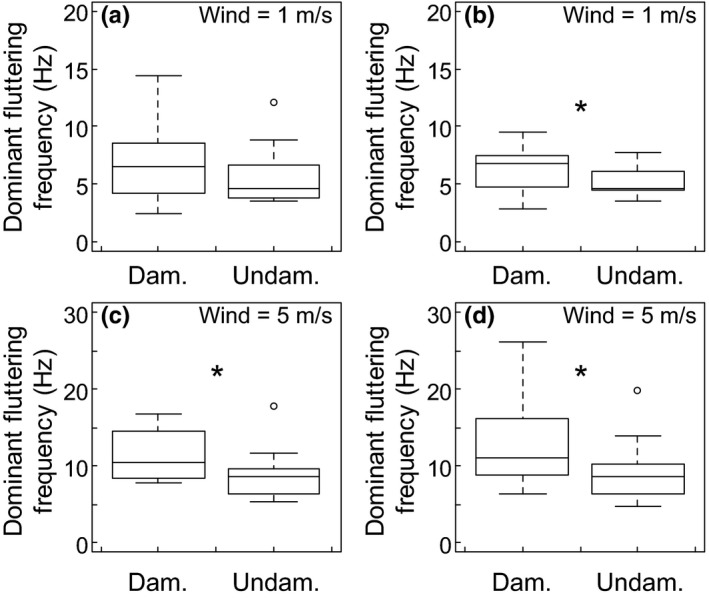
Dominant frequencies of fluttering in the horizontal (a) and vertical (b) directions in 1 m/s wind, and in the horizontal (c) and vertical (d) directions in 5 m/s wind. Asterisks indicate significantly different fluttering frequencies between damaged and undamaged leaves (Mann–Whitney *U*‐tests, *p *< .05)

Herbivory affected the fluttering frequencies of leaves. In 1 m/s wind, there were no differences in the dominant frequencies of horizontal fluttering between damaged and undamaged leaves (Fig. 7a), but damaged leaves did exhibit a faster dominant frequency (median = 6.76 Hz) of vertical fluttering than did undamaged leaves (median = 4.65 Hz) (Fig. 7b). In 5 m/s wind, damaged leaves fluttered at a faster dominant frequency (median = 10.42 Hz) in the horizontal direction than did undamaged leaves (median = 8.67 Hz) (Fig. 7c). Also in 5 m/s wind, the dominant frequency of vertical fluttering was faster for damaged leaves (median = 11.01 Hz) than undamaged leaves (median = 8.57 Hz) (Fig. 7d). Herbivory had no effect on the mean frequencies of horizontal fluttering in either wind speed or for vertical fluttering in 1 m/s wind (Mann–Whitney *U*‐test, *p *>* *.05). However, in 5 m/s wind, the mean frequency of vertical fluttering was faster for damaged leaves (median = 36.58 Hz) than for undamaged leaves (median = 26.50 Hz) (Mann–Whitney *U*‐test, *p *<* *.05). Lastly, the dominant frequencies of fluttering in the horizontal and vertical directions increased for all leaves, regardless of herbivory, when the wind speed increased from 1 to 5 m/s (paired *t* tests, *p *<* *.05, *df *= 33). The mean frequencies of fluttering in the horizontal direction did not change for damaged or undamaged leaves when the wind speed increased (paired *t* test, *p *>* *.05, *df *= 33). The mean frequencies of vertical fluttering increased for damaged leaves, but not undamaged leaves, when the wind speed increased from 1 to 5 m/s (paired *t* test, *p *<* *.05, *df *= 13).

## 4 DISCUSSION

Our data show that invertebrate herbivores can modify a plant's interaction with wind by acting as point masses on the leaves and by removing area from the leaves. The masses of herbivores, or of nonherbivorous animals using leaves for habitat (e.g., Combes, Salcedo, Pandit, & Iwasaki, 2013; Mira & Bernays, 2002), mostly interfered with the orientation and reconfiguration of leaves, whereas herbivore damage (i.e., removal of leaf area) increased the frequency of leaf fluttering. The effect of herbivore mass was more pronounced at the slower wind speed (1 m/s) while that of herbivore damage was more pronounced at the faster wind speed (5 m/s). In total, herbivores can change the way that leaves interact with wind, and thus influence the physical and physiological state of the plants (e.g., Roden & Pearcy, 1993a,b; Vogel, 2009), over a range of wind speeds (1–5 m/s) and through different methods (i.e., adding mass to the leaf or removing leaf area).

The presence of an herbivore on a leaf, before it inflicted any damage to the plant, negatively affected the orientation of the leaf, with heavier herbivore loads causing a greater decrease in pitch (Fig. 3). As predicted, when the herbivore load increased, the combined weight of the herbivore and the leaf produced a twisting moment that caused the leaf to bend about its petiole (Gere & Timoshenko, 1990). Modifying the orientation of the leaf in space can have consequences for the leaf's exposure to solar radiation. For example, when a light source is directly overhead, a leaf whose pitch is largely negative (i.e., well below the horizon) will receive less light per unit surface area than will a perfectly horizontal leaf (King, 1997; Smith & Ullberg, 1989). Therefore, if light is already a limiting factor in photosynthesis, an herbivore's mass on a leaf can further inhibit the plant's growth. Furthermore, changing the leaf's orientation relative to the direction of wind can cause torsional flutter that may increase the mechanical stress on the leaf and petiole (Albayrak et al., 2012).

Large herbivore loads also had a negative effect on the ability of the leaf to reconfigure into a smaller projected area in 1 m/s wind. Reconfiguring into small, streamlined shapes in wind can help reduce the magnitude of aerodynamic forces on the leaf, decreasing the risk of the leaf being damaged or torn from the plant (Gosselin & de Langre, 2009; Vogel, 1989). The negative correlation between herbivore load and reconfiguration suggests that the presence of an herbivore on a leaf may increase the risk of that leaf being damaged or dislodged by forces from the wind. The influence of the herbivore load on reconfiguration was not as prominent in the 5 m/s wind as it was in the 1 m/s wind, further suggesting that aerodynamic forces on the leaf may counteract the effect of the herbivore load once the wind speed is fast enough. Additionally, under faster wind speeds, herbivores on the leaf may be sheltered from the direct force of wind if the leaf reconfigures into a cone that envelops the herbivores, thereby reducing the risk of the herbivores being dislodged from the leaf by aerodynamic forces.

Herbivore loads and their locations on the leaf negatively affected the fluttering frequencies of the leaves, as predicted by simplified models that represent the fluttering leaf as an oscillating beam (French, 1971; Macho‐Stadler et al., 2015). Reduced fluttering frequencies may have consequences for the physiology of the whole plant. Fluttering is an important motion for the leaf because it helps thin the boundary layer of air at the surface of the leaf (Schuepp, 1993), thereby promoting the exchange of gases between the leaf and the surrounding air and helping the leaf lose excess heat, but also increasing water loss through evaporation (Parlange & Waggoner, 1972; Smith & Ennos, 2003; Stokes, Morecroft, & Morison, 2006). Increased gas exchange and proper thermoregulation of the leaf can increase the photosynthetic rate of the plant, but excessive water loss can reduce photosynthetic rates (Roden & Pearcy, 1993b; c; Smith & Jarvis, 1998). That is, herbivores on the leaf can change the photosynthetic rate of the plant just by altering the leaf's interaction with the moving air. This pattern was present in the 1 and 5 m/s wind speeds, suggesting that aerodynamic forces may not counteract the effect of the herbivore in fast wind, as seen with leaf reconfiguration. However, the fluttering frequency that is critical for adequate gas exchange and heat loss may be slow enough that an herbivore load does not necessarily impact the physiology of the plant as long as the leaf can flutter to some small degree (e.g., Roden & Pearcy, 1993a).

Damage from herbivory, effectively decreasing the mass of the leaf, caused leaves to flutter at faster speeds and frequencies, also in accordance with the simplified fluttering leaf model. While faster fluttering can promote gas exchange and heat loss, as described above, it can also alter the light environment within the plant canopy any time the canopy experiences a gust of wind, potentially affecting the photosynthesis and growth of neighboring leaves and plants. More specifically, faster fluttering frequencies can cause the leaf and its neighboring leaves to encounter vastly different light environments on spatial and temporal scales. Intermittent periods of light, known as sunflecks, can influence the photosynthetic rate and growth of plants (Chazdon, 1988; Roden & Pearcy, 1993a; Way & Pearcy, 2012). Faster fluttering frequencies caused by herbivore damage will reduce the duration of each sunfleck, which may elevate the photosynthetic rates of the leaves above the level that would occur in constant light (Adams, Muller, Cohu, & Demmig‐Adams, 2013; Chazdon, 1988; Ögren & Sundin, 1996). On the other hand, damaged leaves have less surface area, and any photosynthetic gain from fluttering faster may be counteracted by the reduced amount of photosynthetic tissue.

The increased fluttering frequency of a damaged leaf can also put additional mechanical stress on the petiole and facilitate abscission of the leaf. Additional mechanical stress on the petiole, from rapidly bending back and forth, can weaken the petiole and cause the damaged leaf to be more easily torn from the tree by drag. This premature dislodgement may act in concert with the tree actively abscising a damaged leaf, which can occur because damaged leaves can be physiologically costly to heal after damage occurs, and because wounds make the plant susceptible to disease (Boege, 2005; Gómez et al., 2012; León, Rojo, & Sánchez‐Serrano, 2001). Loss of damaged leaves by increased fluttering frequencies, in addition to abscission, can help the whole plant conserve nutrients and water for the remaining intact leaves (Blundell & Peart, 2000; Ostlie & Pedigo, 1984).

While we found that presence of herbivores on leaves, and the damage the herbivores inflict on the leaves, can alter the interactions of the leaves with wind, little is known about the influence of plant motion on the herbivores and other animals on the leaf. Recent work found that herbivores avoided plants that were moving (Warren, 2015) or moved off of plants that began moving in wind (Leonard, McArthur, & Hochuli, 2016), but most studies of plant–herbivore interactions do not consider movement by the plant. On the other hand, much work has been done on the neurophysiology and flight biomechanics of flying animals, especially pollinators, that must navigate around moving plants (Mountcastle, Alexander, Switzer, & Combes, 2016) or track the movement of the plant (e.g., the flower) as it moves (Sprayberry & Daniel, 2007). For a crawling herbivore, changes to a leaf's motion in wind, such as increasing the fluttering frequency, could prevent the herbivore from moving on to the leaf, or even dislodge the herbivore if the acceleration of the leaf's fluttering overcome the herbivore's attachment strength to the leaf. Interestingly, the direction of leaf movement became more horizontal as wind speed increased (Table 1), suggesting that an herbivore on a leaf in fast wind might experience horizontal motion and forces, whereas an herbivore on a leaf in slow wind might experience more vertical motion and forces. As a result, modes of attachment or even kinematics of locomotion of a crawling herbivore might vary with wind speed and leaf motion. Overall, the behavioral and physiological responses of herbivores to plant motion in wind remains an open question.

The simultaneous interactions of wind, plants, and herbivores have only recently been considered in ecological studies (Leonard et al., 2016; Warren, 2015). In many instances, ecological studies ignore wind, whereas biomechanical studies of plants in wind ignore the multitude of morphological and physiological changes to the plant that are caused by biological interactions, such as herbivory. In the present study, we show how the simplified case of an herbivore resting on a leaf, and even the damage the herbivore inflicts upon the leaf, can alter that leaf's interaction with wind, and we argue that the altered leaf motion in wind can have many physical and physiological consequences for the plant. Many more aspects of the interactions among plants, animals, and wind have yet to be examined, especially for animals that modify leaf shape (e.g., Labandeira, Wilf, Johnson, & Marsh, 2007), such as galling (Raman, 2011), leaf folding (Loeffler, 1996), and leaf mining (Faeth, 1986; Pincebourde & Casas, 2006).

## AUTHORS' CONTRIBUTIONS

NPB conceived the ideas and designed methodology; ARK and NPB collected and analyzed the data; ARK and NPB led the writing of the manuscript. All authors contributed critically to the drafts and gave final approval for publication.

## CONFLICT OF INTEREST

None declared.

## DATA ACCESSIBILITY

Data are available through the Dryad Digital Repository: https://doi.org/10.5061/dryad.9jg34.
