# Supplementary material for: Herbivores alter plant–wind interactions by acting as a point mass on leaves and by removing leaf tissue
Source: Ecol Evol. 2017 Jul 27;7(17):6884–93. doi: 10.1002/ece3.3249 (PMC5587486; doi:10.1002/ece3.3249)
Supplement: Supplementary file 1 [file ECE3-7-6884-s001.docx]

**Supporting Information for “Herbivores alter plant-wind interactions by acting as a point mass and by removing leaf tissue”**

Adit R. Kothari ^a^, Nicholas P. Burnett ^*a^

^a^ Department of Integrative Biology, University of California, Berkeley, CA 94720, USA

* Corresponding author: burnettnp@berkeley.edu

**Figure S1.** Position of a leaf tip in 5 m s^-1^ wind in the horizontal (a), vertical (b), and net (c) directions. **
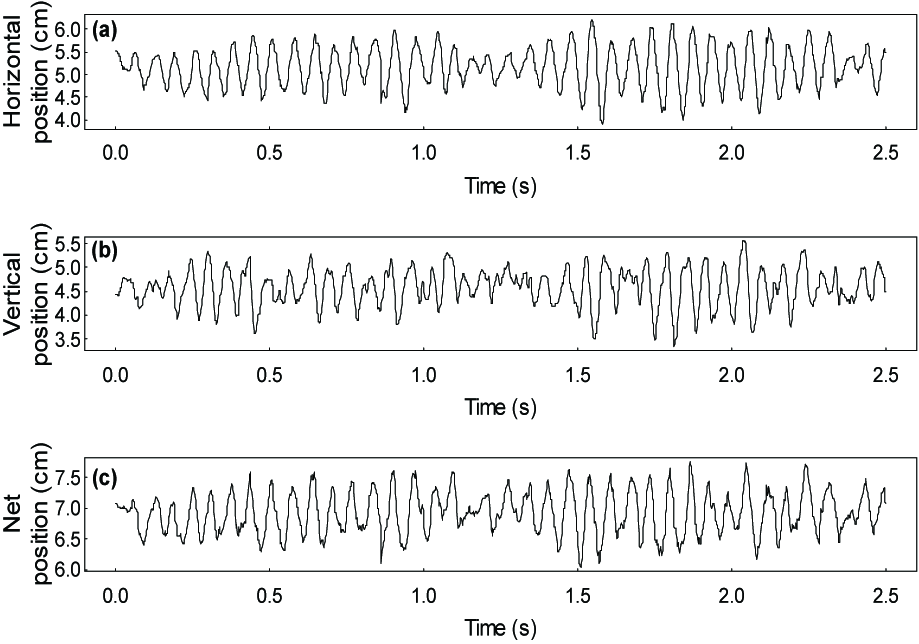
**

**
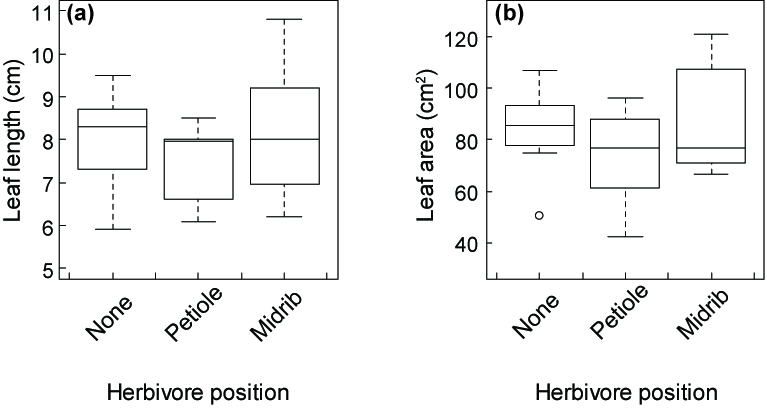
**

**Figure S2.** Leaf lengths (a) and areas (b) of leaves used in the herbivore position experiment. There were no differences in length or area among the three treatments (Kruskal-Wallis tests, *p* > 0.05, d.f. = 2, n = 7 for each group).


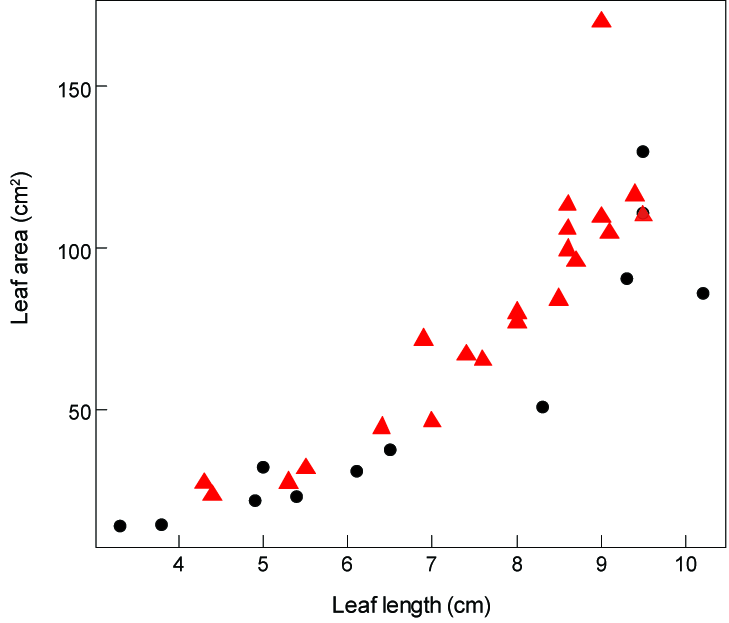


**Figure S3.** Leaf lengths and areas of leaves with naturally-occurring damage from herbivory (black circles) and undamaged leaves (red triangles).

| **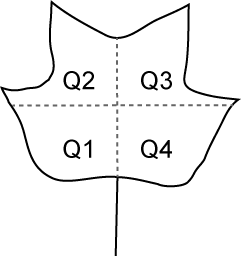** | **Damage location** | **Q1** | **Q2** | **Q3** | **Q4** | **Hole** | **Margin** |
| --- | --- | --- | --- | --- | --- | --- | --- |
|  | **Leaves with damage (%)** | 73.3 | 66.7 | 60.0 | 46.7 | 33.3 | 100.0 |

**Table S1.** Locations of herbivore damage on leaves used in the *Herbivore damage* experiments (n = 15). The area of each leaf was divided into quadrants and scored for the presence of herbivore damage. Leaves were also scored for the presence of herbivore damage that occurred as holes (i.e. missing area that was not at the edge of the leaf) or as margin damage (i.e. missing area that approached the edge of the leaf).
